# geessbin: an R package for analyzing small-sample binary data using modified generalized estimating equations with bias-adjusted covariance estimators

**DOI:** 10.1186/s12874-024-02368-2

**Published:** 2024-11-13

**Authors:** Ryota Ishii, Tomohiro Ohigashi, Kazushi Maruo, Masahiko Gosho

**Affiliations:** 1 Department of Biostatistics, Institute of Medicine, University of Tsukuba, 1-1-1 Tennodai, Tsukuba, 305-8575 Ibaraki Japan; 2 Department of Information and Computer Technology, Faculty of Engineering, Tokyo University of Science, 6-3-1 Niijuku, Katsushika-ku, 125-8585 Tokyo Japan

**Keywords:** Bias correction, Sandwich covariane estimator, Small-sample size

## Abstract

**Background:**

The generalized estimating equation (GEE) method is widely used for analyzing longitudinal and clustered data. Although the GEE estimate for regression coefficients and sandwich covariance estimate are consistent regardless of the choice of covariance structure, they are generally biased for small sample sizes. Various researchers have proposed modified GEE methods and covariance estimators to handle small-sample bias.

**Results:**

We briefly present bias-corrected and penalized GEE methods, along with 11 bias-adjusted covariance estimators. In addition, we focus on analyzing longitudinal or clustered data with binary outcomes using the logit link function and introduce package geessbin in R to implement conventional and modified GEE methods with bias-adjusted covariance estimators. Finally, we illustrate the implementation and detail a usage example of the package. The package is available from the Comprehensive R Archive Network (CRAN) at https://cran.r-project.org/web/packages/geessbin/index.html.

**Conclusions:**

The geessbin package provides three GEE estimates with numerous covariance estimates. It is useful for analyzing correlated data such as longitudinal and clustered data. Additionally, the geessbin is designed to be user-friendly, making it accessible to non-statisticians.

**Supplementary Information:**

The online version contains supplementary material available at 10.1186/s12874-024-02368-2.

## Background

The generalized estimating equation (GEE) method is widely used to analyze longitudinal and clustered data, and it provides consistent estimates of the regression coefficients even if the working correlation structure is misspecified. In addition, the covariance estimator for the regression coefficients called the sandwich covariance estimator, is consistent and robust with respect to misspecification of the working covariance structure [1]. However, the GEE estimate for regression coefficients is biased in small sample sizes regardless of the specified working correlation structure [2]. Furthermore, the sandwich covariance estimator is downwardly biased in small samples, even when the working correlation structure is correctly specified [3]. These biases lead to invalid statistical inferences for the regression coefficients.

To prevent biased estimator of regression coefficients from the GEE method, Paul and Zhang [2], Lunardon and Scharfsten [4], and Mondol and Rahman [5] proposed bias-adjusted GEE methods for small sample sizes. Although Paul and Zhang [2] first proposed a bias-corrected estimator of regression coefficients, the estimator relies on the correct specification of the working correlation structure. Lunardon and Scharfsten [4] provided a modified bias-corrected estimator that is valid even when the working correlation structure is misspecified. This method is known as the bias-corrected GEE (BCGEE) method. Its analysis can be conducted using the R package BCgee. Mondol and Rahman [5] proposed the penalized GEE (PGEE) method for analyzing longitudinal data when the outcome is binary. The PGEE method uses a Firth-type penalty to reduce the bias of the regression coefficients in the presence of separation. PGEE method can be conducted using the geefirth function in the R package geefirthr.

To address the small-sample bias of sandwich covariance estimates, numerous modified covariance estimators have been proposed in the GEE setting [3, 6–13]. In addition, Lu et al. [14], Wang et al. [15], Gosho et al. [16], Ford and Westgate [17], and Gosho et al. [18] reviewed these estimators and compared their performance through simulation studies. The covariance estimates proposed by Mancl and DeRouen [3], Kauermann and Carroll [6], Fay and Graubard [7], Pan [8], Morel et al. [9], Wang and Long [10], Fan et al. [11], and Gosho et al. [12] can be calculated using the R package geesmv.

While geesmv package is useful for analyzing small-sample longitudinal and clustered data, it does not include the recent modified covariance estimators and cannot implement the BCGEE and PGEE methods. Similarly, BCgee and geefirthr support only one type of covariance estimators. Hence, in existing packages, researchers have to analyze small-sample data with limited combinations of modified GEE methods and adjusted covariance estimators.

Recently, Gosho et al. [19] reviewed the BCGEE and PGEE and compared the performance of the modified GEE methods with the conventional method through a simulation study. They also applied eight modified covariance estimators with the three GEE methods. Based on Gosho et al. [19], we additionally consider three modified covariance estimators and present a new R package geessbin that implements 11 covariance estimation methods adjusted for small sample bias in the GEE, BCGEE, and PGEE methods. The proposed package offers practitioners an easily implementable tool to analyze small-sample clustered or longitudinal data with any combination of modified GEE methods and covariance estimators. The package was developed in the Microsoft Windows environment and requires R 3.5.0 or later. We briefly describe the available modified covariance estimators in Modified covariance estimators section.

## Implementation

### GEE and modified GEE methods

Let $n_i$ be the number of observations in the *i*-th cluster ($i=1,\ldots ,K$). $X_i=(X_{i1},\ldots ,X_{in_i})^\top$ and $Y_i=(Y_{i1},\ldots ,Y_{in_i})^\top$ denote the covariate and outcome vectors for the *i*-th cluster, respectively. We assume that $\mu _{it}$ is expected value $\textrm{E}(Y_{it}\mid X_{it})$ for $t = 1, \ldots , n_i$ and $i = 1, \ldots , K$ and expressed as function $\mu _{it}=h^{-1}(X_{it}^\top \beta )$ of the linear predictor through link function *h*, where $\beta$ is a *p*-dimensional regression coefficient vector. The variance of $Y_{it}$ is assumed to be $\textrm{Var}(Y_{it}\mid X_{it})=\phi \upsilon (\mu _{it})$ with scale parameter $\phi$ and variance function $\upsilon$. Let $R_i(\alpha )$ be a specified working correlation matrix parameterized by $\alpha$. Then, the covariance matrix of $Y_i$ is expressed as $V_i = \phi A_i^{1/2}R_i(\alpha )A_i^{1/2}$, where $A_i=\textrm{diag}(\upsilon (\mu _{it}))$.

#### Conventional GEE method

The GEE method identifies the estimator $\hat{\beta }$ of the regression coefficient $\beta$ as the solution to the following estimating equation, substituting $\phi$ with $K^{1/2}$-consistent estimator $\hat{\phi }(Y,\beta )$ after replacing $\alpha$ with $K^{1/2}$-consistent estimator $\hat{\alpha }(Y,\beta ,\phi )$.

1 $$\begin{aligned} U = U(\beta ) = \sum \limits _{i=1}^{K}D_i^\top V_i^{-1} \varepsilon _i=0, \end{aligned}$$

where $D_i$ is an $n_i \times p$ matrix defined by $D_i=D_i(\beta )=\partial \mu _i/\partial \beta ^\top$, $\varepsilon _i=Y_i-\mu _i$, and $\mu _i=(\mu _{i1},\ldots ,\mu _{in_i})^\top$. The Fisher information matrix is defined by $\Phi =\sum \nolimits _{i=1}^{K} D_i^\top V_i^{-1}D_i$. A detailed explanation of the estimation procedure employed in the geessbin package is provided in the Supplementary Material.

The general form of the covariance matrix of $\hat{\beta }$ is given by

2 $$\begin{aligned} \Phi ^{-1} \left\{ \sum \limits _{i=1}^{K} D_i^\top V_i^{-1} \textrm{Cov} (Y_i) V_i^{-1}D_i\right\} \Phi ^{-1}. \end{aligned}$$

However, this covariance matrix cannot be obtained since $\textrm{Cov} (Y_i)$ in Eq. (2) is unknown. Hence, by replacing $\textrm{Cov} (Y_i)$ with $\hat{\varepsilon }_i\hat{\varepsilon }_i^\top$, the sandwich (robust) covariance estimator of $\hat{\beta }$ is defined as follows:

3 $$\begin{aligned} \mathrm {Cov_{SA}}(\hat{\beta }) = \Phi ^{-1} \left\{ \sum \limits _{i=1}^{K} D_i^\top V_i^{-1} \hat{\varepsilon }_i\hat{\varepsilon }_i^\top V_i^{-1}D_i\right\} \Phi ^{-1}, \end{aligned}$$

where $$\hat{\varepsilon }_i = Y_i - \hat{\mu }_i$$.

$\hat{\beta }$ and $\mathrm {Cov_{SA}}(\hat{\beta })$ have consistency even under misspecification of the working correlation structure [1]. However, these estimators can yield a bias when the sample size is small. For example, Gosho et al. [19] performed simulation studies with small-sample sizes $K = 20$, 30, and 50 and showed the bias of these estimators.

Below, we briefly describe the modified GEE methods. Gosho et al. [19] present more detailed explanations of these methods.

#### BCGEE method

Paul and Zhang [2] proposed a bias-corrected estimator in the context of GEE using the second-order approximation of the estimator. However, their estimator is unsuitable for application because it relies on the correct specification of the working correlation structure. Lunardon and Scharfstein [4] addressed this problem by developing a modified bias-corrected estimator. Let $\kappa _{rs}=\textrm{E}[\partial U_r / \partial \beta _s]$, $\kappa _{r,s} = \textrm{E}[U_rU_s]$, $\kappa _{rsu}=\textrm{E}[\partial ^2 U_r / \partial \beta _s \partial \beta _u]$, $\kappa _{rs,u} = \textrm{E}[(\partial U_r / \partial \beta _s)U_u]$, and $\kappa _{rs}^{(u)}=\partial \kappa _{rs} / \partial \beta _u$ for $r, s, u = 1,\ldots ,p$, where $U_r$ is the *r*-th element of *U*. We assume that $\kappa ^{rs}$ is the (*r*, *s*)-th element of the inverse of the matrix $[\kappa _{rs}]$. The bias of $\hat{\beta }$ derived by Lunardon and Scharfstein is given by

$$\begin{aligned} b_r = \sum \limits _{s = 1}^p \kappa ^{rs}\sum \limits _{u,v = 1}^p\left[ \kappa _{su,v}-\frac{1}{2}\sum \limits _{k,\ell = 1}^p\kappa ^{k\ell }\kappa _{sv\ell }\kappa _{u,k}\right] \kappa ^{uv}, \quad r= 1,\ldots ,p. \end{aligned}$$

Then, the BCGEE estimator is expressed as follows:

$$\begin{aligned} \hat{\beta }_{\textrm{BC}} = \hat{\beta } - (b_1, \ldots , b_p)^\top . \end{aligned}$$

#### PGEE method

Mondol and Rahman [5] addressed a separation problem and small-sample bias of $\hat{\beta }$ by adding a Firth-type penalty term to Eq. (1). Specifically, the PGEE estimate is given by the solution of the following simultaneous equation:

$$\begin{aligned} U_r + \frac{1}{2} \textrm{tr}\left[ \Phi ^{-1} \frac{\partial \Phi }{\partial \beta _r} \right] = 0, \quad r= 1,\ldots ,p. \end{aligned}$$

This equation is solved by using an iterative process as well as the conventional GEE method described in the Supplementary Material.

### Modified covariance estimators

In this section, we present 11 modified covariance estimators. The following eight estimators are described only as mathematical formulae. Gosho et al. [19] provide additional explanations.

- Kauermann and Carroll estimator [6]
  $$\begin{aligned} \mathrm {Cov_{KC}}(\hat{\beta }) = \Phi ^{-1} \left\{ \sum \limits _{i = 1}^K D_i^\top V_i^{-1} (I_i-H_{ii})^{-1/2} \hat{\varepsilon }_i \hat{\varepsilon }_i^\top (I_i-H_{ii}^\top )^{-1/2} V_i^{-1} D_i\right\} \Phi ^{-1} \end{aligned}$$
- Mancl and DeRouen estimator [3]
  $$\begin{aligned} \mathrm {Cov_{MD}}(\hat{\beta }) = \Phi ^{-1} \left\{ \sum \limits _{i = 1}^K D_i^\top V_i^{-1} (I_i-H_{ii})^{-1} \hat{\varepsilon }_i \hat{\varepsilon }_i^\top (I_i-H_{ii}^\top )^{-1} V_i^{-1} D_i\right\} \Phi ^{-1} \end{aligned}$$
- Fay and Graubard estimator [7]
  $$\begin{aligned} \mathrm {Cov_{FG}}(\hat{\beta }) = \Phi ^{-1} \left\{ \sum \limits _{i = 1}^K F_i D_i^\top V_i^{-1} \hat{\varepsilon }_i \hat{\varepsilon }_i^\top V_i^{-1}D_i F_i \right\} \Phi ^{-1} \end{aligned}$$
- Pan estimator [8]
  $$\begin{aligned} \mathrm {Cov_{PA}}(\hat{\beta })= \Phi ^{-1} \left\{ \sum \limits _{i = 1}^K D_i^\top V_i^{-1}A_i^{1/2} \left( \frac{1}{K} \sum \limits _{j = 1}^K A_j^{-1/2}\hat{\varepsilon }_j \hat{\varepsilon }_j^\top A_j^{-1/2}\right) A_i^{1/2}V_i^{-1}D_i\right\} \Phi ^{-1} \end{aligned}$$
- Gosho et al. estimator [12]
  $$\begin{aligned} \mathrm {Cov_{GS}}(\hat{\beta }) = \frac{K}{K-p} \mathrm {Cov_{PA}}(\hat{\beta }) \end{aligned}$$
- Morel et al. estimator [9]
  $$\begin{aligned} \mathrm {Cov_{MB}}(\hat{\beta }) =\Phi ^{-1} \left\{ \Psi +\gamma_1\gamma_2 \Phi \right\} \Phi ^{-1} \end{aligned}$$
- Wang and Long estimator [10]
  $$\begin{aligned} \mathrm {Cov_{WL}}(\hat{\beta })= \Phi ^{-1} \left\{ \sum \limits _{i = 1}^K D_i^\top V_i^{-1} A_i^{1/2} \left( \frac{1}{K} \sum \limits _{j = 1}^K A_j^{-1/2}(I_j-H_{jj})^{-1} \hat{\varepsilon }_j \hat{\varepsilon }_j^\top (I_j-H_{jj}^\top )^{-1} A_j^{-1/2} \right) A_i^{1/2} V_i^{-1}D_i\right\} \Phi ^{-1} \end{aligned}$$
- Westgate and Burchett estimator [13]
  $$\begin{aligned} \mathrm {Cov_{WB}}(\hat{\beta }) = \Phi ^{-1} \left\{ \sum \limits _{i = 1}^K D_i^\top V_i^{-1} A_i^{1/2} \left( \frac{1}{K} \sum \limits _{j = 1}^K A_j^{-1/2}(I_j-H_{jj})^{-1/2} \hat{\varepsilon }_j \hat{\varepsilon }_j^\top (I_j-H_{jj}^\top )^{-1/2} A_j^{-1/2} \right) A_i^{1/2} V_i^{-1}D_i\right\} \Phi ^{-1} \end{aligned}$$

The notation for the above estimators is as follows. $H_{ij}=D_i \Phi ^{-1} D_j^\top V_j^{-1}$. $I_i$ is an identity matrix of the same dimension as $H_{ii}$. $F_i$ is a diagonal matrix $[F_i]_{ss} = \{1 - \min (\delta , [N_i]_{ss})\}^{-1/2}$. $N_i = D_i^\top V_i^{-1} D_i \Phi ^{-1}$, $d_i = D_i^\top V_i^{-1}\hat{\varepsilon }_i$, and $\bar{d} = \sum \nolimits _{i=1}^K d_i/K$. Ψ is a *p* × *p* matrix defined as

$$\mathrm{\Psi}=\frac{\sum^{K}_{i=1}\;n_i-1}{\sum^{K}_{i=1}\;n_i-p}\frac{K}{K-1}\sum_{i=1}^{K}\left(d_i-\overline d\right)\left(d_i-\overline d\right)^{\mathrm T}.$$

γ_1_ = min (0.5, *p*/(*K *- *p*)) and γ_2_ = max (1, tr (Φ^-1^ Ψ)/*p*).

In addition to the eight abovementioned estimators, we consider the three estimators described below.

- MacKinnon and White estimator [20]
  $$\begin{aligned} \mathrm {Cov_{MK}}(\hat{\beta }) = \frac{K}{K-p}\mathrm {Cov_{SA}}(\hat{\beta }). \end{aligned}$$
  $\mathrm {Cov_{MK}}(\hat{\beta })$ clearly approaches $\mathrm {Cov_{SA}}(\hat{\beta })$ as $K \rightarrow \infty$.
- Ford and Wastgate estimator [17]
  $$\begin{aligned} \mathrm {Cov_{FW}}(\hat{\beta }) = \frac{1}{2}\left\{ \mathrm {Cov_{KC}}(\hat{\beta }) + \mathrm {Cov_{MD}}(\hat{\beta })\right\} . \end{aligned}$$
- Fan et al. estimator [21]
  $$\begin{aligned} \mathrm {Cov_{FZ}}(\hat{\beta }) = \Phi ^{-1}\left\{ \sum \limits _{i=1}^KD_i^\top V_i^{-1}(I_i - H_i)^{-1}\left( \hat{\varepsilon }_i\hat{\varepsilon }_i^\top - \sum \limits _{j \ne i}H_{ij}\hat{\varepsilon }_j\hat{\varepsilon }_j^\top H_{ij}^\top \right) ((I_i - H_i)^\top )^{-1}V_i^{-1}D_i\right\} \Phi ^{-1}. \end{aligned}$$

Recently, Gosho et al. [19] evaluated the performance of the eight modified covariance estimators (above estimators except $\mathrm {Cov_{MK}}(\hat{\beta })$, $\mathrm {Cov_{FW}}(\hat{\beta })$, and $\mathrm {Cov_{FZ}}(\hat{\beta })$) with the three GEE methods. Although it is difficult to conclude which modified covariance estimator is the best with limited simulation studies, their simulation results shows that the Morel et al. estimator yields the smallest bias among adjusted covariance estimators in many settings. However, in some scenarios of their simulation study, Morel et al. estimator is conservative and another covariance estimator is preferred.

### Description of the geessbin function

In this section, we describe the geessbin function in the geessbin package considering an example dataset. The package is available from the Comprehensive R Archive Network (CRAN) and can be installed and loaded using the following code:
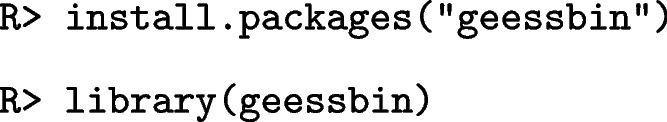


The dataset used in this study was reported in Hardin and Hilbe [22]. The data studied the effect of air pollution on the health of 16 children. The outcome variable was the wheezing status measured consistently four times yearly at ages of 9, 10, 11, and 12 years. This dataset contains five variables, as detailed in Table 1.

**Table 1:** Variable definitions in wheeze dataset

Variable	Description
ID	Child identifier
Wheeze	Binary indicator of wheezing presence
City	Binary indicator of whether the child lives in Kingston
Age	Age of child in years ranging from 9 to 12
Smoke	Measure of smoking habits of child’s mother

The observations of the first two children are as follows:
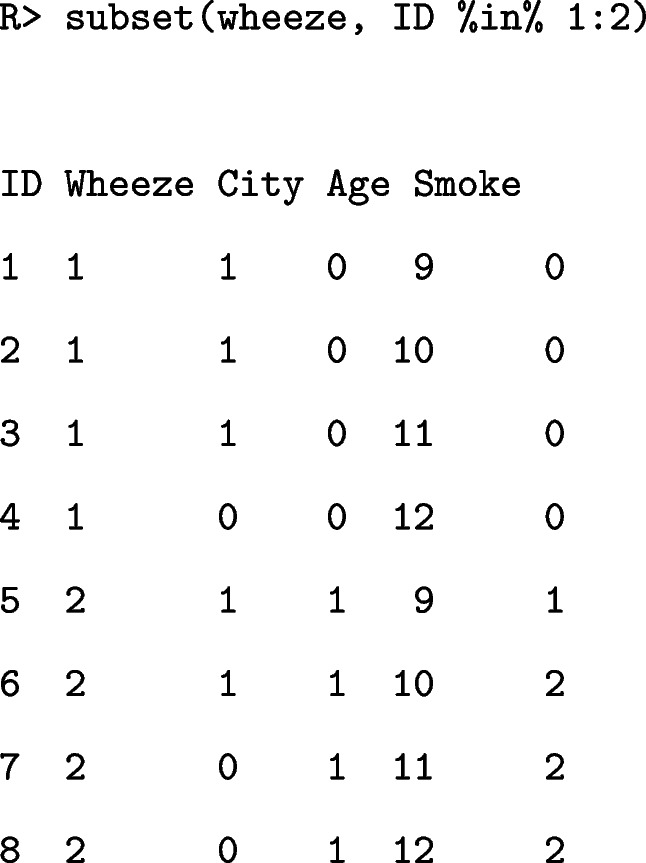


## Results

The geessbin function provides any combination of three GEE methods (GEE, BCGEE, and PGEE) and 12 covariance estimators (unadjusted estimator $\mathrm {Cov_{SA}}(\hat{\beta })$ and 11 adjusted estimators described in above). This function supports only binomial outcomes with the logit link. The arguments are listed in Table 2. The usage of geessbin is similar to that of the existing functions gee and geeglm, with the exception of the arguments repeated, beta.method, and SE.method.

**Table 2:** Arguments of the geessbin function

Argument	Description
formula	Object of class formula: symbolic description of model to be fitted (see documentation of lm and formula for details
data	Data frame containing variables used in model
id	Numeric or character vector that identifies the subjects or clusters (NULL by default)
repeated	Numeric or character vector that identifies repeatedly measured variable within each subject or cluster. This variable is treated as a categorical variable in order to construct a correlation matrix. If repeated = NULL (default value), as is the case in function gee, data are assumed to be sorted so that observations on a cluster are contiguous rows for all entities in the formula.
corstr	Working correlation structure. The following are permitted: "independence", "exchangeable", "ar1", and "unstructured" ("independence" by default).
beta.method	Method for estimating regression parameters. The following are permitted: "GEE", "PGEE", and "BCGEE" ("PGEE" by default).
SE.method	Method for estimating standard errors. The following are permitted: "SA", "MK", "KC", "MD", "FG", "PA", "GS", "MB", "WL", "WB", "FW", and "FZ" ("MB" by default).
b	Numeric vector specifying initial values of regression coefficients. If b = NULL (default value), the initial values are calculated using the ordinary or Firth logistic regression assuming that all the observations are independent.
maxitr	Maximum number of iterations (50 by default)
tol	Tolerance used in fitting algorithm ($10^{-5}$ by default)
scale.fix	Logical variable; if TRUE, the scale parameter is fixed at 1 (FALSE by default)
conf.level	Numeric value of confidence level for confidence intervals (0.95 by default)

We can specify the estimation methods for regression coefficients and standard errors in beta.method and SE.method arguments, respectively. The repeated argument is used to identify the time points in longitudinal data analysis. On the other hand, the repeated argument is used to specify the subject identifier within clusters in clustered data analysis. The vector in the repeated argument does not need to be equal in length for each cluster; hence, clustered data with different cluster sizes are acceptable.

The geessbin function returns a list of class geessbin. As with glm and gee functions, we can apply print, summary, vcov, coef, residuals, model.matrix, and fitted functions to returned objects. The list includes the components for the modified GEE with bias-adjusted covariance estimators (Table 3).

**Table 3:** Values of the geessbin function

Component	Description
call	Matched call
coefficients	Named numeric vector of regression coefficients
linear.predictors	Numeric vector of linear predictors
fitted.values	Numeric vector of fitted probabilities
residuals	Numeric vector of residuals
scale	Numeric value of scale parameter
covb	Variance–covariance matrix of regression coefficients
wcorr	Working correlation matrix
iterations	Numeric value of specified maximum number of iterations
beta.method	Character value of specified estimation method for regression coefficients
SE.method	Character value of specified estimation method for standard errors
K	Numeric value of number of subjects or clusters
max.ni	Numeric value of maximum number of observations within subjects or clusters
corstr	Character value of specified working correlation structure
convergence	Character value of convergence status
conf.level	Numeric value of specified confidence level
model.matrix	Design matrix
data	Data frame

The following is an example code for analyzing the wheeze dataset with the following model:

$$\begin{aligned} \log \left( \frac{\textrm{Pr}(\texttt {Wheeze} = 1)}{1-\textrm{Pr}(\texttt {Wheeze} = 1)}\right) = \texttt {City} + \texttt {Age}. \end{aligned}$$

Here, Age is regarded as a categorical variable and AR(1) working correlation structure is used. The PGEE method and Morel et al. estimator $\mathrm {Cov_{MB}}(\hat{\beta })$ are used for estimating the regression coefficients and standard errors, respectively.
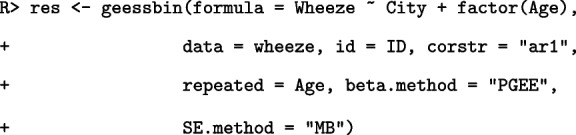


The print function provides information regarding the model details, estimation methods, regression coefficients, scale parameter, number of iterations, working correlation matrix, and convergence status as follows:
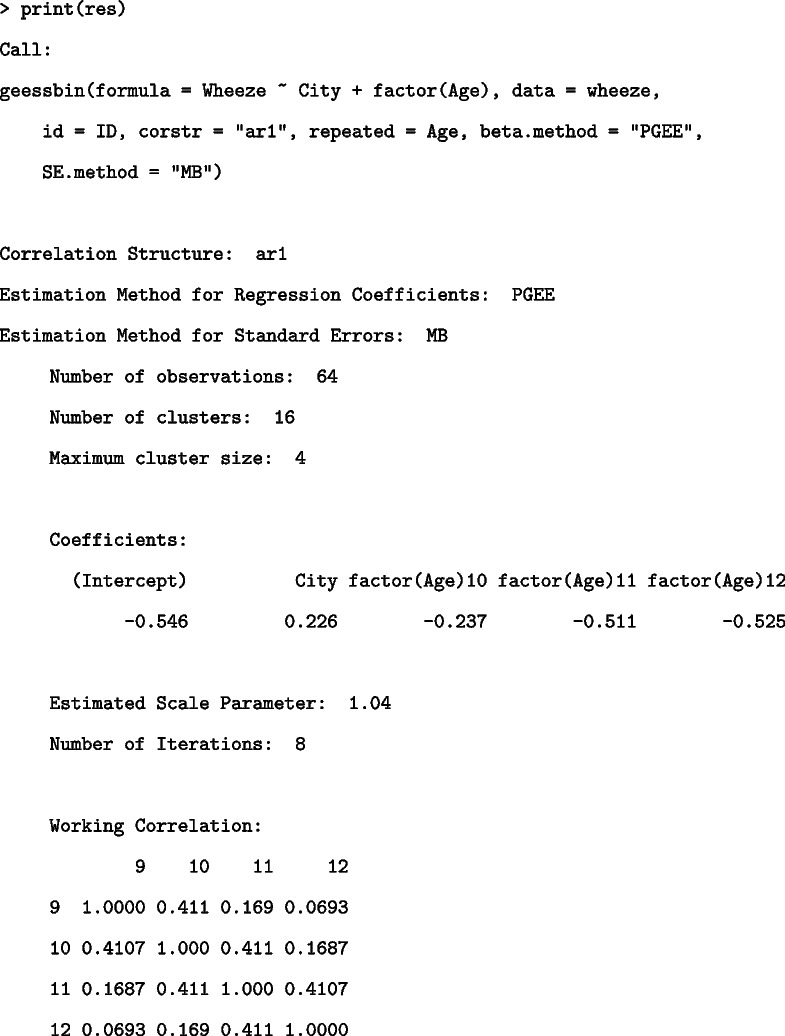


The summary function provides the following results of hypothesis tests using the estimated regression coefficients and standard errors and odds ratios with confidence limits.
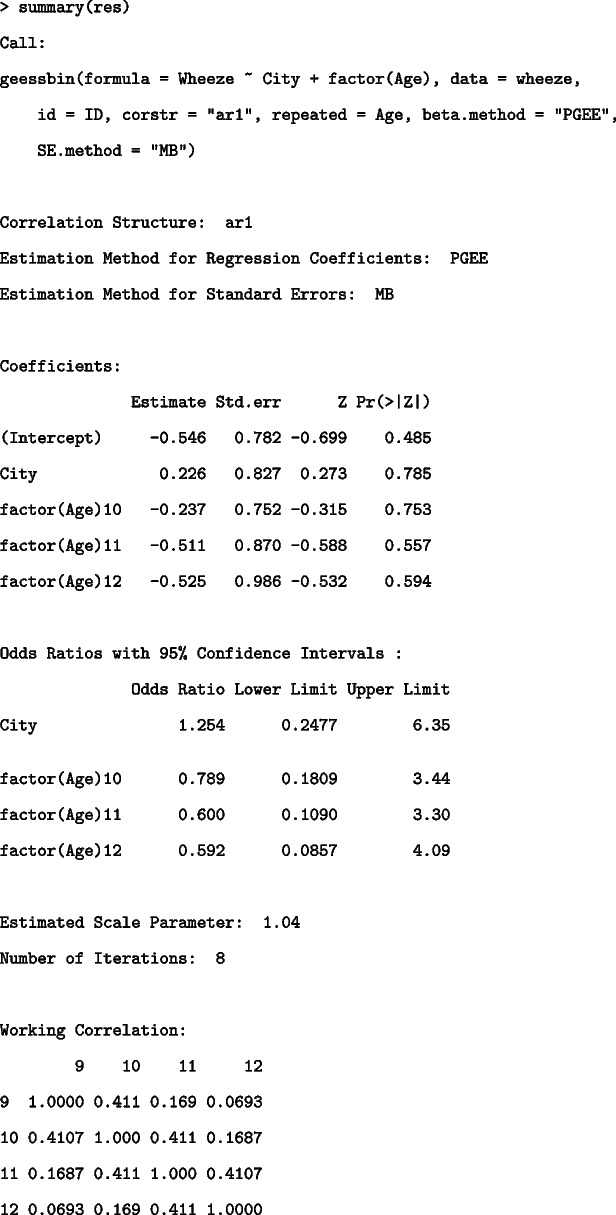


The Coefficients object provides *Z* statistics and two-sided *p*-values for each coefficient. The Odds Ratios with 95% Confidence Intervals object lists the odds ratios and confidence intervals. The confidence intervals are calculated as exponents of $\text {(Estimates of regression coefficient)}\pm z_{\alpha / 2}\times \text {(Standard error of the estimates of regression coefficient)}$, where $z_{\alpha / 2}$ is the $100(1-\alpha / 2)$th percentile of the standard normal distribution. The two objects in the above results indicated that all the variables were not significant in this model and there was a tendency for the odds to decrease with increasing age.

The geessbin_all function can be used to obtain the results from all combinations with the following code: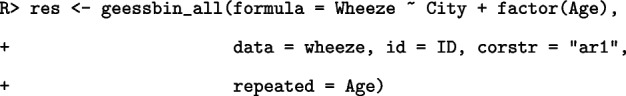 For example, all combinations of three GEE methods and 12 covariance estimators for the regression coefficient $\beta _{\texttt {City}}$ for City are listed in Table 4.

**Table 4:** Parameter estimates and its standard errors for the regression coefficient $\beta _{\texttt {City}}$ of City in wheeze dataset

	GEE	BCGEE	PGEE
Estimates of $\beta _{\texttt {City}}$	0.265	0.270	0.226
Standard errors			
SA	0.647	0.629	0.622
MK	0.780	0.758	0.750
KC	0.692	0.672	0.665
MD	0.740	0.719	0.711
FG	0.719	0.699	0.691
PA	0.651	0.633	0.625
GS	0.785	0.763	0.754
MB	0.863	0.841	0.827
WL	0.744	0.723	0.714
WB	0.696	0.676	0.668
FW	0.716	0.696	0.688
FZ	0.698	0.678	0.671

The usage of the geessbin_all function is largely similar to the geessbin function, but it does not require the beta.method and SE.method arguments. The geessbin_all function returns a list with two tables. The first table provides regression coefficients, standard errors, *z* statistics, and *p*-values, whereas the second provides odds ratios and confidence intervals.

## Conclusion

We developed the geessbin package and demonstrated the use of the geessbin function, which implements all combinations of three GEE methods (GEE, BCGEE, and PGEE) and 12 covariance estimators (unadjusted estimator $\mathrm {Cov_{SA}}(\hat{\beta })$ and 11 adjusted estimators). This function offers practitioners a flexible tool to analyze small-sample clustered or longitudinal data with binary outcomes. Recently, Gosho et al. [19] evaluated the performance of each combination of the modified GEE methods and eight bias-adjusted covariance estimators through simulation studies. Hence, their simulation results may be helpful when choosing a combination in practice.

The geessbin function has limitations. This function assumes binary outcomes and does not support count outcomes. The reason is that the PGEE method for the Poisson distribution is not developed in existing studies although the BCGEE method supports continuous and count outcomes. In addition, this function supports only the logit link function. We did not consider the covariance inflation proposed by Westgate [23, 24], because we focused on adjustments of the small-sample bias. We will expand the package to address these limitations in future work.

## Supplementary Information

Supplementary Material 1.

## Data Availability

The dataset used in this study was reported in Hardin and Hilbe (2013). Hardin, J. W., & Hilbe, J. M. (2013). Generalized estimating equations. chapman and hall/CRC.

## References

[1] Liang KY, Zeger SL. Longitudinal data analysis using generalized linear models. Biometrika. 1986;73:13–22. 10.1093/biomet/73.1.13.

[2] Paul S, Zhang X. Small sample GEE estimation of regression parameters for longitudinal data. Stat Med. 2014;33:3869–81. 10.1002/sim.6198. 24797886 10.1002/sim.6198

[3] Mancl LA, DeRouen TA. A covariance estimator for GEE with improved small-sample properties. Biometrics. 2001;57:126–34. 10.1111/j.0006-341X.2001.00126.x. 11252587 10.1111/j.0006-341x.2001.00126.x

[4] Lunardon N, Scharfstein D. Comment on ‘Small sample GEE estimation of regression parameters for longitudinal data’. Stat Med. 2017;36:3596–600. 10.1002/sim.7366. 28868672 10.1002/sim.7366

[5] Mondol MH, Rahman MS. Bias-reduced and separation-proof GEE with small or sparse longitudinal binary data. Stat Med. 2019;38:2544–60. 10.1002/sim.8126. 30793784 10.1002/sim.8126

[6] Kauermann G, Carroll RJ. A note on the efficiency of sandwich covariance matrix estimation. J Am Stat Assoc. 2001;96:1387–96. 10.1198/016214501753382309.

[7] Fay MP, Graubard BI. Small-sample adjustments for Wald-type tests using sandwich estimators. Biometrics. 2001;57:1198–206. 10.1111/j.0006-341X.2001.01198.x. 11764261 10.1111/j.0006-341x.2001.01198.x

[8] Pan W. On the robust variance estimator in generalised estimating equations. Biometrika. 2001;88:901–6. 10.1093/biomet/88.3.901.

[9] Morel JG, Bokossa MC, Neerchal NK. Small sample correlation for the variance of GEE estimators. Biom J. 2003;45:395–409. 10.1002/bimj.200390021.

[10] Wang M, Long Q. Modified robust variance estimator for generalized estimating equations with improved small-sample performance. Stat Med. 2011;30:1278–91. 10.1002/sim.4150. 21538453 10.1002/sim.4150

[11] Fan C, Zhang D, Zhang CH. A comparison of bias-corrected covariance estimators for generalized estimating equations. J Biopharm Stat. 2013;23:1172–87. 10.1080/10543406.2013.813521. 23957522 10.1080/10543406.2013.813521

[12] Gosho M, Sato Y, Takeuchi H. Robust covariance estimator for small-sample adjustment in the generalized estimating equations: A simulation study. Sci J Appl Math Stat. 2014;2:20–5. 10.11648/j.sjams.20140201.13.

[13] Westgate PM, Burchett WW. Improving power in small-sample longitudinal studies when using generalized estimating equations. Stat Med. 2016;35:3733–44. 10.1002/sim.6967. 27090375 10.1002/sim.6967 PMC4965318

[14] Lu B, Preisser JS, Qaqish BF, Suchindran C, Bangdiwala SI, Wolfson M. A comparison of two bias-corrected covariance estimators for generalized estimating equations. Biometrics. 2007;63:935–41. 10.1111/j.1541-0420.2007.00764.x. 17825023 10.1111/j.1541-0420.2007.00764.x

[15] Wang M, Kong L, Li Z, Zhang L. Covariance estimators for generalized estimating equations (GEE) in longitudinal analysis with small samples. Stat Med. 2016;35:1706–21. 10.1002/sim.7131. 26585756 10.1002/sim.6817 PMC4826860

[16] Gosho M, Hirakawa A, Noma H, Maruo K, Sato Y. Comparison of bias-corrected covariance estimators for MMRM analysis in longitudinal data with dropouts. Stat Methods Med Res. 2017;26:2389–406. 10.1177/0962280215597938. 26265765 10.1177/0962280215597938

[17] Ford WP, Westgate PM. A comparison of bias-corrected empirical covariance estimators with generalized estimating equations in small-sample longitudinal study settings. Stat Med. 2018;37:4318–29. 10.1002/sim.7917. 30073684 10.1002/sim.7917

[18] Gosho M, Noma H, Maruo K. Practical review and comparison of modified covariance estimators for linear mixed models in small-sample longitudinal studies with missing data. Int Stat Rev. 2021;89:550–72. 10.1111/insr.12447.

[19] Gosho M, Ishii R, Noma H, Maruo K. A comparison of bias-adjusted generalized estimating equations for sparse binary data in small-sample longitudinal studies. Stat Med. 2023;42:2711–27. 10.1002/sim.9744. 37062288 10.1002/sim.9744

[20] MacKinnon JG, White H. Some heteroskedasticity-consistent covariance matrix estimators with improved finite sample properties. J Econ. 1985;29:305–25. 10.1016/0304-4076(85)90158-7.

[21] Fan C, Zhang D, Zhang CH. Robust small-sample inference for fixed effects in general Gaussian linear models. J Biopharm Stat. 2012;22:544–64. 10.1080/10543406.2011.557792. 22416840 10.1080/10543406.2011.557792

[22] Hardin JW, Hilbe JM. Generalized Estimating Equations. 2nd ed. London: Chapman and Hall; 2013. 10.1201/b13880.

[23] Westgate PM. A bias correction for covariance estimators to improve inference with generalized estimating equations that use an unstructured correlation matrix. Stat Med. 2013;32:2850–8. 10.1002/sim.5709. 10.1002/sim.5709 23255154

[24] Westgate PM. A covariance correction that accounts for correlation estimation to improve finite-sample inference with generalized estimating equations: a study on its applicability with structured correlation matrices. J Stat Comput Simul. 2016;86:1891–1900. 10.1080/00949655.2015.1089873. 10.1080/00949655.2015.1089873 PMC5089177 27818539

