# Supplementary material for: geessbin: an R package for analyzing small-sample binary data using modified generalized estimating equations with bias-adjusted covariance estimators
Source: BMC Med Res Methodol. 2024 Nov 13;24:277. doi: 10.1186/s12874-024-02368-2 (PMC11558877; doi:10.1186/s12874-024-02368-2)
Supplement: Supplementary file 1 — Supplementary Material 1. [file 12874_2024_2368_MOESM1_ESM.pdf]

Supplementary Material for "geessbin: An R package for analyzing small-sample binary data using modified generalized estimating equations with bias-adjusted covariance estimators"

Ryota Ishii, Tomohiro Ohigashi, Kazushi Maruo, and Masahiko Gosho

GEE procedure in `geessbin`

We provide the detailed procedure of the generalized estimating equation (GEE) estimation employed in the `geessbin` package in this Supplementary Material.

Let n_i be the number of observations in the i-th cluster $(i = 1, \ldots, K)$. $X_i = (X_{i1}, \ldots, X_{in_i})^\top$ and $Y_i = (Y_{i1}, \ldots, Y_{in_i})^\top$ denote the covariate and outcome vectors for the i-th cluster, respectively. We assume that μ_{it} is expected value $\mathrm{E}(Y_{it} \mid X_{it})$ for $t = 1, \ldots, n_i$ and $i = 1, \ldots, K$ and expressed as function $\mu_{it} = h^{-1}(X_{it}^\top \beta)$ of the linear predictor through link function h, where β is a p-dimensional regression coefficient vector. The variance of Y_{it} is assumed to be $\mathrm{Var}(Y_{it} \mid X_{it}) = \phi \upsilon(\mu_{it})$ with scale parameter ϕ and variance function υ. Let $R_i(\alpha)$ be a specified working correlation matrix parameterized by α. Then, the covariance matrix of Y_i can be expressed as $V_i = \phi A_i^{1/2} R_i(\alpha) A_i^{1/2}$, where $A_i = \mathrm{diag}(\upsilon(\mu_{it}))$.

The GEE method identifies the estimator $\hat{\beta}$ of the regression coefficient β as the solution to the following estimating equation, substituting ϕ with $K^{1/2}$-consistent estimator $\hat{\phi}(Y, \beta)$ after replacing α with $K^{1/2}$-consistent estimator $\hat{\alpha}(Y, \beta, \phi)$.

$$U = U(\beta) = \sum_{i=1}^{K} D_i^\top V_i^{-1} \varepsilon_i = 0, \tag{1}$$

where D_i is an $n_i \times p$ matrix defined by $D_i = D_i(\beta) = \partial \mu_i / \partial \beta^\top$, $\varepsilon_i = Y_i - \mu_i$, and $\mu_i = (\mu_{i1}, \ldots, \mu_{in_i})^\top$. The Fisher information matrix is defined by $\Phi = \sum_{i=1}^{K} D_i^\top V_i^{-1} D_i$. Estimator $\hat{\beta}$ is calculated using the following iterative process:

(i) Let $\beta^{(0)}$ be an initial value of β and k be 0.

(ii) Calculate scale parameter ϕ as

$$\hat{\phi} = \frac{\sum_{i=1}^{K} \sum_{t=1}^{n_i} \hat{r}_{it}^2}{\sum_{i=1}^{K} n_i - p},$$

where $\hat{r}_{it} = (Y_{it} - h^{-1}(X_{it}^\top \beta^{(k)})) / \sqrt{\upsilon(X_{it}^\top \beta^{(k)})}$.

(iii) Calculate the working correlation matrix as follows. If the working correlation structure is exchangeable, that is, $\mathrm{Cor}(Y_{it}, Y_{it'}) = \alpha$ for all $t \neq t'$,

$$\hat{\alpha} = \frac{\sum_{i=1}^{K} \sum_{t<t'} \hat{r}_{it}\hat{r}_{it'}}{\hat{\phi}\left\{\sum_{i=1}^{K} n_i(n_i-1)/2 - p\right\}}.$$

If the working correlation structure is first-order autoregressive (AR(1)), that is, $\mathrm{Cor}(Y_{it}, Y_{it'}) = \alpha^{|t-t'|}$ for all $t \neq t'$,

$$\hat{\alpha} = \frac{\sum_{i=1}^{K} \sum_{t=1}^{n_i-1} \hat{r}_{it}\hat{r}_{i(t+1)}}{\hat{\phi}\left\{\sum_{i=1}^{K}(n_i-1) - p\right\}}.$$

If the working correlation structure is unstructured, that is, $\mathrm{Cor}(Y_{it}, Y_{it'}) = \alpha_{tt'}$ for all $t \neq t'$,

$$\hat{\alpha}_{tt'} = \frac{\sum_{i=1}^{K} \hat{r}_{it}\hat{r}_{it'}}{\hat{\phi}(K-p)}.$$

(iv) Calculate U in Eq (1) using $\beta^{(k)}$ and the above estimates. If $\max(|U_1|, \ldots, |U_p|)$ is less than a prespecified tolerance, finish the iterative process and the define estimate $\hat{\beta}$ by $\beta^{(k)}$.

(v) Update current value $\beta^{(k)}$ as $\beta^{(k+1)} = \beta^{(k)} + \Phi^{-1}U$ and iterate steps (ii)–(iv), where Φ is calculated using $\beta^{(k)}$ and the abovementioned estimates.
